# Natural Language Processing Analysis of WhatsApp Messages to Identify Unrecognized HIV Prevention Needs Among Young Women in Kenya

**DOI:** 10.1002/jia2.70214

**Published:** 2026-10-06

**Authors:** Jessica E. Haberer, Bernard Rono, Rakesh Ravi, Vinal Bagaria, Sinchana Shetty, Shanmukh Moparthi, Kevin Oware, Josephine B. Odoyo, Lindsey Garrison, William Rand, Elizabeth A. Bukusi

**Affiliations:** ^1^ Center for Global Health Massachusetts General Hospital Boston Massachusetts USA; ^2^ Department of Medicine Harvard Medical School Boston Massachusetts USA; ^3^ Centre for Microbiology Research Kenya Medical Research Institute Kisumu Kenya; ^4^ North Carolina State University Raleigh North Carolina USA; ^5^ Departments of Obstetrics and Gynecology and Global Health University of Washington Seattle Washington USA

**Keywords:** HIV prevention, natural language processing, sub‐Saharan Africa, young women

## Abstract

**Introduction:**

Identifying HIV prevention needs is challenging. Common approaches (e.g. public health campaigns, HIV risk scores) have limited sensitivity. Natural language processing techniques, such as topic modelling and computational linguistics, can extract meaning and subjective information from text. This study used these techniques with WhatsApp messages to identify unrecognized HIV prevention needs among young women in Kenya.

**Methods:**

Prior work established key ethical protections (e.g. privacy, confidentiality). Between July and December 2024, women (18−24 years) who used WhatsApp were enrolled from four clinical sites in Kisumu, Kenya. After completing a socio‐demographic questionnaire, participants securely transferred all WhatsApp messages from the prior 6 months and provided anonymized relationship types (e.g. friend, sexual partner) for their 20 most frequent contacts. Data were de‐identified, and only outgoing messages were retained; all photos and incoming messages were deleted. WhatsApp data were pre‐processed using a downloaded large language model to translate Swahili and Dholuo to English and account for slang linguistic patterns. We explored multiple approaches to topic modelling in the WhatsApp data using latent Dirichlet allocation. Non‐negative matrix factorization and uniform manifold approximation and projection allowed for comparison of participant word topics with the VOICE HIV risk score (range 0–8, 8 indicating high risk).

**Results:**

Of 413 eligible young women, 13 (3%) declined to provide WhatsApp data, and 22 had insufficient data for analysis. Participants had a mean age of 22 years and a VOICE risk score of 5.7. The remaining 378 women provided a median of 14 chats per individual. Participants most commonly chatted with friends (62.4%), followed by sexual partners (18.1%). The median VOICE risk score was 7 for women whose most messaged relationship was a sexual partner versus 5 for those messaging a friend most often. Topic models had differential correlation with the VOICE risk score that was most strongly positive (0.12) when indicating romance/affection and most strongly negative (−0.09) when describing faith/transactions. Dimensional analysis of linguistic similarity identified discrete participant populations with distinct VOICE risk scores.

**Conclusions:**

This study provides proof‐of‐concept that natural language processing of routine WhatsApp messages can yield meaningful individual‐level information about HIV prevention needs. Future work should compare such findings with HIV incidence to optimally determine its ability to identify unrecognized HIV prevention needs, thus potentially expanding HIV prevention services to individuals not currently engaged in care.

## 1 Introduction

Numerous HIV prevention interventions exist today that could make elimination of new HIV acquisitions a reality if used effectively by individuals at risk for HIV exposure, including HIV pre‐exposure prophylaxis (PrEP) [1, 2, 3, 4], condoms, reduced number of sexual partners, male circumcision and harm reduction measures for people who inject drugs [5]. The impact of these interventions is greatest when their use is aligned with risk of HIV exposure—a concept known as prevention‐effective adherence [6]. However, knowledge of HIV risk can be challenging for many individuals, thus resulting in missed opportunities for HIV prevention.

Numerous tools are used for HIV risk assessment, such as normative guidelines and risk calculators or scores [7, 8, 9]; however, all have limited ability to identify individuals with HIV prevention needs, particularly when applied to large populations. For example, young women in sub‐Saharan Africa (SSA) account for 63% of all new acquisitions on the continent, yet risk varies greatly within that population of >70 million women [10, 11]. A well‐known tool, the VOICE risk score, was developed from three HIV prevention trials in African women (VOICE, HPTN 035 and FEM‐PrEP) with high levels of predictive ability and rigorous internal and external validation [7]. However, in practice, this tool has performed sub‐optimally. For instance, application of the VOICE risk score produced only moderate areas under the curve (0.58−0.73) when applied to the Evidence for Contraceptive Options and HIV Outcomes Trial [12]. Similarly, limited performance was seen in a systematic review of risk‐based screening tools to optimize HIV testing services [13]. While relatively effective for identifying acute HIV among men who have sex with men (MSM) in high‐income settings, the authors urged caution “when using risk‐based screening tools for other populations.” Moreover, risk assessment can be stigmatizing and may limit engagement in HIV prevention, particularly for young women [14]. The concept of “risk” may also not resonate with many people, especially youth who may focus more on developmentally appropriate goals, such as forming relationships and addressing practical demands (e.g. education) [15]. Given these challenges, new approaches to identifying HIV prevention needs are needed to complement traditional services, such as the development of passive tools that analyse existing information (e.g. smartphone data) and automatically provide insights into HIV‐related behaviours and social circumstances using data science methods.

One such approach is natural language processing (NLP), which involves techniques like topic modelling and computational linguistics to extract meaning and subjective information from text [16]. NLP of text and social media messages can provide insights into human behaviour and has potential to determine the risk of HIV exposure. NLP has been successfully applied to understand probability in multiple contexts. For instance, short message service (SMS) texts have been used to predict micro‐loan repayment better than standard financial background checks [17]. Also, topic modelling has been used to survey and map HIV and COVID transmission using Twitter data [18, 19], and NLP has been applied to clinical HIV screening [20] and prediction of sexual and substance use behaviour in the United States [21]. It has not, however, been applied to understanding HIV prevention needs through SMS or social media in SSA.

NLP can typically be performed in minutes and thus could enable real‐time prediction of HIV prevention needs and large‐scale deployment as a smartphone app. Notably, smartphone usage is widespread globally, including in low‐ and middle‐income settings. For example, >80% of Kenyans aged 18–34 years own mobile phones [22]. Smartphones are also increasing in prevalence and were estimated to account for almost two‐thirds of connections in SSA in 2025 [23]. Automated analysis of text and social media is thus a promising, practical tool that should be explored for guiding HIV prevention needs.

Ethical principles must be considered carefully when collecting and analysing text and social media messages, particularly regarding privacy and confidentiality [24]. However, as HIV prevention needs are not being met through existing approaches, there is an ethical justification to consider the inherent risks versus the potential benefits. These issues must be considered not only from the perspective of the individual owning the messages, but also from contacts involved in two‐way communications (i.e. “bystanders” [25]).

Here, we present a pilot NLP analysis of WhatsApp messages among young women in Kenya. After conducting in‐depth ethical preparatory work, we explored several approaches to understanding the use of multiple languages, topic modelling, messaging patterns and correlation with currently used HIV risk assessment tools based on questionnaire data. This study provides proof‐of‐concept for this novel approach to understanding HIV prevention needs.

## 2 Methods

### 2.1 Study Setting

This study was based at the Kenyan Medical Research Institute Research Care and Training Program (KEMRI RCTP) in Kisumu, Kenya. We recruited participants from Lumumba Sub‐County Hospital, Kisumu County Hospital, Kuoyo Health Center Sub‐County Hospital and Ahero County Hospital, which are <1, 2, 3 and 22 km from RCTP, respectively.

### 2.2 Ethical Considerations

Prior to performing the NLP analysis presented here, we conducted a qualitative study with 32 young Kenyan women (half who would and half who would not hypothetically provide WhatsApp messages), as well as nine Kenyan bioethicists, to explore ethical concerns. Topics covered classical domains (e.g. privacy, confidentiality, autonomy, beneficence and non‐maleficence) as well as contextual concerns (e.g. neocolonial power, justice) [26]. Results are presented elsewhere [27]. Briefly, participants were most concerned about privacy, use of third‐party messages, potential for secondary consequences (e.g. detection of illegal activity) and uncertainty around data interpretation. Identified mitigation strategies included multiple layers of data anonymization and clear explanations prior to data use (e.g. analysis of words, not sentences, eliminated potential detection of illegal activity). These mitigation strategies were implemented in the quantitative study presented in this paper, and all activities were compliant with the Kenya Data Protection Act [28, 29]. Study procedures were approved by the institutional review boards at KEMRI and Mass General Brigham.

### 2.3 Community Sensitization

Prior to study initiation, study team members gave public talks around the four clinic recruitment sites. We additionally held a meeting with a Kisumu‐based Community Advisory Board to discuss any concerns and identify facilitators for study implementation. All recommendations were included in the study protocol.

### 2.4 Enrolment

We recruited participants between July and December 2024 by approaching young women presenting to the four above‐noted clinics for any health services, including PrEP and HIV testing. Inclusion criteria were: age 18–24 years; smartphone ownership (indicating sole usage); and ability to understand Swahili, DhoLuo and/or English (the most spoken languages in the region). The only exclusion criterion was inability to provide informed consent (e.g. intoxication, developmental delay). All participants were asked for informed verbal consent to provide socio‐demographic information. Eligible and interested participants were offered informed consent for full study participation in a private location using their preferred language.

### 2.5 Data Collection

A study research assistant administered a brief questionnaire on socio‐demographic information and comfort with the collection of anonymized WhatsApp messages. She then retrospectively downloaded all the participants’ WhatsApp messages from the prior 6 months (no messages were selectively censored or deleted to avoid social desirability bias) on a password‐protected laptop dedicated to the study. All sync services to cloud apps were disabled prior to data transfer. As all participants used Android phones, data transfer took place via a temporary Bluetooth connection in a private location (>10 m from other devices), thus ensuring other phones could not access the data. Intentional device pairing was confirmed for each participant with a unique code, and media transfer was deactivated at the point of data transfer. Transferred data was stored initially on the study laptop using a unique participant identifier code without the participant's name or other identifiable information; phone numbers were reduced to the final five digits. Python scripts were used to systematically delete all incoming messages and convert the time/date of the message into a date/month only format for additional data protection. Participants also provided a list of anonymized types of relationships (e.g. friend, sexual partner, family) for up to 20 people most frequently contacted via WhatsApp; no names were collected, and all relationships were labelled by the last five digits of their phone numbers to enable anonymized matching with the participant's WhatsApp messages. Anonymized data was then uploaded to an encrypted DropBox folder for analysis. Additionally, participants were offered point‐of‐care HIV testing if it was not already done at the clinic that day, after the completion of other data collection so as not to bias the findings; individuals testing positive were linked to appropriate services.

### 2.6 Message Processing

Multiple large language models (LLMs) were explored for translation to English from Swahili, Dhuluo and/or any combination of these languages (e.g. Sheng); ultimately, a downloaded version of Llama3 was used, which prevented any external access to the data. Stop words (e.g. “the,” “in”), repeated phrases and system messages were removed as they contribute minimal meaning to the analysis. Emojis were also removed because their meaning was not consistently clear with available context. Lemmatization (i.e. reducing words to their base form) was applied using SpaCy to normalize word forms. Ad hoc review of translations by multi‐lingual study staff was conducted when clustered words were not intuitively related.

### 2.7 Analysis of Socio‐Demographic Data

Participant characteristics were analysed descriptively. The VOICE risk score was calculated as: age <25 (2 points), being single/not living with a primary sexual partner (2 points), lacking financial/material support from a sexual partner (1 point), a sexual partner having/potentially having other partners (2 points) and alcohol use (1 point) [4]. We also calculated the Wand risk score as: age (<20 years, 11 points; 20–24 years, 10 points; 25–29 years 6 points, 30+ years, 0 points), sexual debut <16 years old (3 points), not being married/living with a partner (13 points), having children (0−1 children, 10 points; 2 children, 8 points; 3+ children, 0 points), using injectable contraception (4 points), 3+ sex partners in past 3 months (5 points) and ever diagnosed with a sexually transmitted infection (STI) (4 points) [7]. Scores of >5 and ≥25 suggest high risk (HIV incidence >5/100 person‐years) with the VOICE and Wand risk scores, respectively.

### 2.8 Topic Modelling

We explored the WhatsApp dataset using topic modelling (i.e. analysis of word clusters, or “topics”) using latent Dirichlet allocation (LDA), which works well for short text clustering [30], as well as with simple textual counts. Analysis also involved tokenization (i.e. turning the words into tokens with core meanings). The LDA model was trained using multiple iterations of individual‐level “bag of words” to generate 10 words per topic. The model averaged topic distributions and assigned a dominant topic per participant. We also created models based on context‐driven topics with the use of “seeds,” or select words, to see if text clusters existed around those words. For example, “sex” might be related to increased HIV risk, while “amen” might not (see Appendix A1). We also used non‐negative matrix factorization, which produces latent clusters of topics and indicates how strongly topics associate with each other.

### 2.9 Model Evaluation

We assessed our models according to homogeneity, completeness, V‐measure, adjusted Rand index, normalized mutual information and adjusted mutual information [30, 31]. We also explored linear versus multidimensional modelling.

### 2.10 Embedding and Dimensionality Reduction

Each participant's pre‐processed and cleaned dataset (including text content and multiple HIV risk characteristics) was converted into dense numerical representations (called embeddings) using the all‐MiniLM‐L6‐v2 model [32]. This technique enables analysis of semantic similarity, which may be underappreciated with topic modelling and is particularly important in contexts using informal language or slang. The embedding approach also enables concurrent assessment of similarity in language with similarity in HIV risk scores. Uniform manifold approximation and projection was then applied to reduce high‐dimensional embeddings into a two‐dimensional space, preserving both the local and global structure of relationships among embeddings [33]. This approach maximizes both correlation and difference; in other words, similar populations regarding language use and HIV prevention needs would appear close together, whereas different populations would appear farther apart. Clustering was performed using k‐means clustering (i.e. minimizing the distance between each data point and the averaging of its assigned cluster), which segments participants into groups based on linguistic similarity. This approach was used to expose potential patterns in conversational behaviour that might align with HIV risk indicators.

### 2.11 Comparison With Questionnaire‐Based HIV Risk Scores

We used Pearson correlations to assess the VOICE and Wand risk scores with selected word topics and the most frequently contacted relationship type, including timing of communications.

## 3 Results

### 3.1 Participant Characteristics

We screened 698 young women, of whom 298 were not eligible because of age <18 or >24 years (*N* = 98), no smartphone ownership (*N* = 110), no WhatsApp use (*N* = 77) and lack of willingness to share messages (*N* = 13). We enrolled the remaining 400 young women (100 per site). See Table 1 for participant characteristics. Two participants were found to have HIV.

**TABLE 1:** Participant characteristics of enrolled and ineligible participants.

	Study participants (*N* = 400)	Ineligible participants (*N* = 298)	
Characteristic	*N* (%) or mean (SD)	*N* (%) or mean (SD)	*p*‐value
**Demographics**			
Average age (in years; SD)	22.0 (1.6)	23.7 (4.2)	<0.001
Education (in years)	13.4 (1.3)	12.3 (1.8)	<0.001
Residence type			<0.001
Urban	90 (23%)	41 (14%)	
Semi‐urban	282 (71%)	193 (65%)	
Rural	28 (7%)	64 (21%)	
Marital status			<0.001
Single, no steady partner	161 (40%)	67 (22%)	
Single, steady partner	174 (44%)	82 (28%)	
Married, one wife	61 (15%)	128 (43%)	
Married, multiple wives	3 (<1%)	20 (7%)	
Divorced/widowed	1 (<1%)	1 (<1%)	
Living arrangement			<0.001
Alone	82 (21%)	41 (14%)	
With parents/other family	200 (50%)	93 (31%)	
With spouse/sexual partner	77 (19%)	162 (54%)	
With friends	41 (10%)	2 (1%)	
Any alcohol use in the past 3 months			<0.001
Yes	90 (23%)	16 (5%)	
No	310 (78%)	280 (95%)	
**Sexual behaviour**			
Average number of sexual partners in the past 3 months (SD)	2.5 (2.4)	1.4 (0.8)	<0.001
Average at sexual debut (in years; SD)	17.8 (1.9)	17.6 (2.1)	0.20
Partner provides financial/material support			0.34
Yes	314 (79%)	245 (83%)	
No	62 (16%)	35 (12%)	
No current partner	20 (5%)	16 (5%)	
Missing/not sexually active	4 (1%)	2 (<1%)	
Partner has other sexual partners			0.13
Yes	128 (32%)	82 (28%)	
No	57 (14%)	62 (21%)	
Not sure	191 (48%)	136 (46%)	
No current partner	20 (5%)	16 (5%)	
Missing/not sexually active	4 (1%)	2 (<1%)	
Use of any family planning currently			1.00
Yes	130 (33%)	97 (33%)	
No	266 (67%)	199 (67%)	
Missing/not sexually active	4 (1%)	2 (<1%)	
Number of children			<0.001
0	279 (70%)	145 (49%)	
1	116 (29%)	111 (37%)	
2+	5 (1%)	42 (14%)	
History of known STI			0.25
Yes	33 (8%)	18 (6%)	
No	359 (90%)	276 (93%)	
Missing	4 (1%)	2 (<1%)	
Average VOICE risk score (SD)	5.7 (1.4)	4.1 (1.9)	<0.001
Average Wand risk score (SD)	32.8 (6.4)	25.5 (8.6)	<0.001

Abbreviations: SD, standard deviation; STI, sexually transmitted infection.

Compared to study participants, we found significant differences for ineligible participants, including age (as expected per eligibility criteria), lower education, more rural residence type, more married participants, more living arrangements involving a spouse/sexual partner, less alcohol use, fewer sexual partners in the past 3 months, more children, and lower VOICE and WAND HIV risk scores. No significant differences were found for age at sexual debut, partner support, partner concurrency or family planning use.

When asked, “How do you feel about providing us with your messages?”, enrolled participants answered: very comfortable (142; 36%), comfortable (201; 50%), neutral (18; 5%), a little uncomfortable (4; 1%) and not comfortable at all (0; 0%). The consent form was reviewed with the four participants who reported “a little uncomfortable”; all confirmed a desire to participate in the study.

### 3.2 WhatsApp Dataset

Twenty‐two participants had insufficient data for analysis; the remaining 378 women provided a median of 14 chats (interquartile range [IQR] 11–20) with unique individuals with a median of 478 words (IQR 198–894), for a total of 6607 files. Chats lasted a median of 20 days (IQR 5–100); see Figure A1 for visual representations of these descriptive statistics. Language composition included English, Swahili, Sheng (mixture of English and Kiswahili) and Dholuo.

### 3.3 Social Interactions and HIV Risk Scores

No comparisons of the most messaged relationship and the VOICE risk score were statistically distinct, as shown in Figure 1 (see Figure A2 for similar comparisons with the Wand risk score). However, participants whose most messaged relationship was a “sexual partner” had the highest median VOICE risk score; the distribution of scores in this group was homogeneously high, potentially reflecting behavioural or psychosocial indicators associated with sexual relationships. Other participants had more spread in their scores, implying mixed risk patterns, perhaps influenced by diverse message content (social vs. emotional).

**FIGURE 1:**
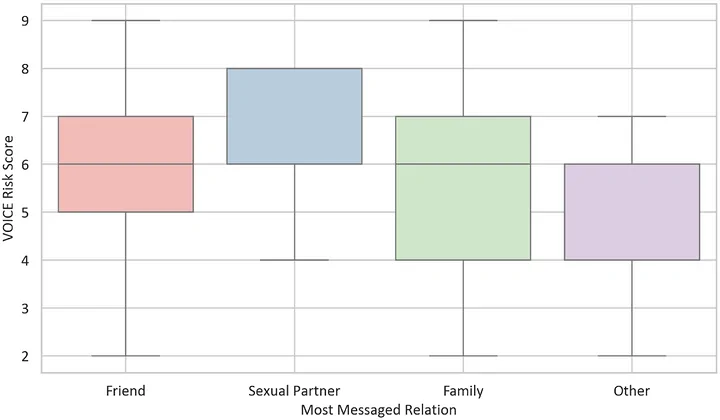
Distribution of the VOICE risk score by most messaged relationship (i.e. friend, sexual partner, family member or other).

### 3.4 Word Representations

Numerous word groupings from all participants’ WhatsApp data were generated and reviewed for potential correlation with the VOICE and Wand HIV risk scores. Figure 2 provides an example of a word cloud from a romance‐related LDA topic identified from the overall dataset.

**FIGURE 2:**
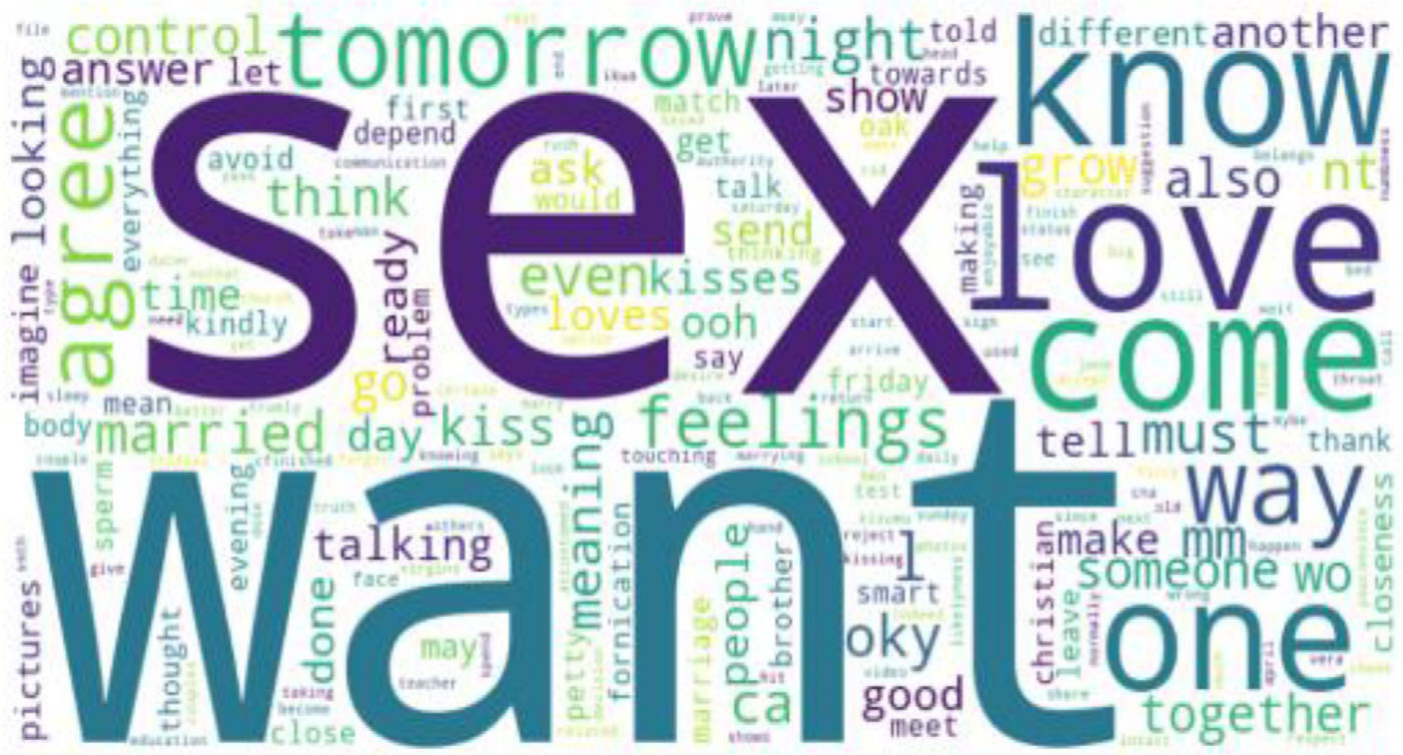
Example of a word cloud of WhatsApp messages (larger size indicates more frequent use of the word).

### 3.5 Correlation of Word Topics with HIV Risk Scores

Single context‐driven word tokens and selected unidimensional modelling provided limited correlation with the HIV risk scores; however, seeded word clusters and multidimensional modelling were more revealing. As shown in Figure 3, topic models had variable correlation with the VOICE risk score (see Figure A3 for the Wand risk score analysis, which is similar). Topics 2 and 4 (romance/affection) were associated with higher VOICE risk scores, while topics 6 (faith/positivity) and 8 (financial transaction/family) were associated with lower VOICE risk scores, potentially representing stable social contexts.

**FIGURE 3:**
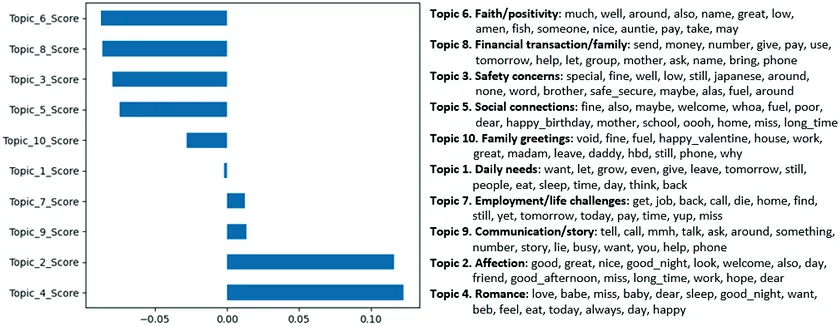
Non‐negative matrix factorization showing correlation between sample topics and the VOICE HIV risk score.

Figure 4 shows uniform manifold approximation and projection visualization of MiniLM text embeddings. This visualization indicates how participants’ linguistic embeddings (derived purely from WhatsApp content) related to their VOICE risk scores (seeFigure A4 for similar analysis with the Wand risk score). The dimensional analysis does not have physical meaning but preserves semantic similarity; in essence, participants close together have similar conversational language patterns. Yellow‐green clusters (higher risk) appear in localized regions, suggesting some linguistic styles were associated with higher risk behaviours. Blue‐purple regions (lower risk) dominate elsewhere, representing participants whose chats contained less risk‐linked language (e.g. non‐sexual communication). The absence of strict boundaries means language use and risk overlapped, and many participants exhibited blended conversational cues. This figure demonstrates that linguistic similarity alone carries a partial signal about behavioural risk. Participants with comparable chat tone and vocabulary tended to cluster together, and their VOICE risk levels varied gradually, not randomly. In other words, risk‐related language was embedded within everyday conversation patterns, not in distinct categories.

**FIGURE 4:**
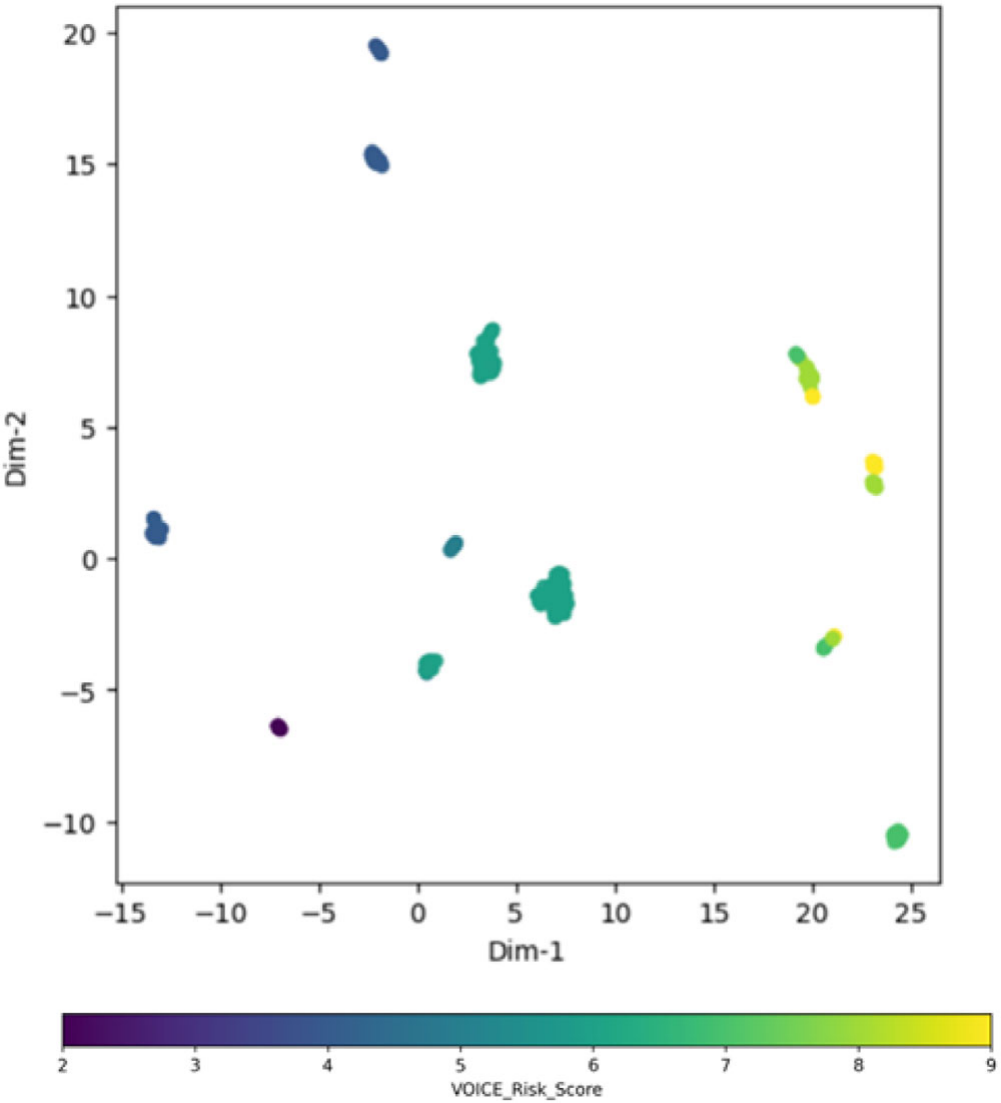
Uniform manifold approximation and projection visualization showing clustering of participants with linguistic similarity as it relates to the VOICE risk score. The axes (Dim [dimension] 1 and 2) are the first two dimensions, which represent a compression of each participant's MiniLM sentence embedding. Purple indicates low risk; yellow indicates high risk.

## 4 Discussion

This study presents a novel use of NLP to understand HIV prevention needs among young women seeking care in Kenya. The high willingness and degree of comfort with providing WhatsApp data indicate openness to this type of analysis. The correlations between topics in the WhatsApp data and questionnaire‐based HIV risk scores demonstrate that meaningful information exists in the data patterns with face validity (e.g. topics on romance/affection correlated with higher risk scores). However, the distinctions among identified topics and participant clusters were not statistically significant, and correlations were modest. This pilot study thus provides proof‐of‐concept for further exploration of this novel approach to identifying HIV prevention needs.

Our study adds to other similarly promising applications of NLP for HIV prevention, which have thus far been confined to the United States. For example, in a recent analysis of MSM, NLP was used to analyse free text responses regarding reasons for discontinuing PrEP [34]. Results identified specific barriers (e.g. relationship issues, insurance concerns) with moderately high‐performance metrics among various algorithm approaches (e.g. support vector machine, random forest, gradient boost). Another study performed word analysis, Unified Medical Language System semantic type analysis and topic modelling on Twitter/X posts [35]. The authors identified key areas of personal discussion (e.g. advocacy, access, stigma) as distinct from promotional information and irrelevant messages.

With any of these analyses, ethical protections are critical. Detailed assessment of the ethical concerns explored and mitigated in this study are published separately [27]. Briefly, key issues include individual privacy and confidentiality concerns and country‐specific data protection laws. The acceptability and feasibility of this approach are illustrated by the fact that 97% of eligible young women provided their WhatsApp data and felt comfortable/very comfortable doing so. Many aspects of data science are becoming ever more commonplace, and NLP is likely to be applied to myriad contexts. Our thorough preparatory work and the corresponding application of mitigation strategies sets a standard for broader applications in health‐related research. That said, additional ethical protections and transparency on data use will be critical when applying these tools outside controlled research contexts.

The data analytics in this study reflect a preliminary evaluation; further exploration could increase the ability to identify HIV prevention needs. Importantly, the VOICE and Wand risk scores are imperfect tools. A study with HIV incidence as the comparator would be ideal for further assessment, which can now be considered for future research given our proof‐of‐concept findings. Other promising analyses include incorporating additional preprocessing steps to remove noise and normalize linguistic structures, as well as annotating key behavioural and emotional indicators in collaboration with Kenyan youth and public health experts. In this current work, we only used an LLM for translation. Using an LLM for the analysis could allow for the model to better take into account the entire conversation history of an individual. A specifically trained LLM (potentially using open‐weight tuning based on contextual knowledge) could enable direct prediction from the data to the risk score, thus avoiding common challenges found with translation. To enhance performance and accuracy, hybrid and ensemble models that combine LLM‐derived predictions with traditional features, such as those explored in this paper, may be beneficial. Additionally, we analysed this data with LDA given its utility for topic discoverability and interpretable themes in exploratory analyses, and focused on the word level, given limitations of LLM‐based translation from multiple languages, with consequent reliance on preprocessing and tokenization to reduce noise and protect interpretability. Future work with larger datasets and improved translation models should add bidirectional encoder representations from transformers (BERT), which can retain conversational context and improve data classification, topic modelling and sentiment analysis.

A key application of this analysis is to expand the HIV prevention cascade more proximally. That is, our current understanding of HIV prevention needs often starts with people who undergo HIV testing. However, many people may be at risk without recognizing the need for testing. Development of an NLP‐based app could enable widespread use. The anticipated volume of data could likely be run in 10 min either within healthcare systems or the community, and a downloadable version could be run natively on a smartphone to protect privacy and confidentiality. Moreover, information about HIV prevention needs based on passively collected data (i.e. WhatsApp messages) avoids social desirability and other barriers to health‐seeking, such as stigma. Similar work to expand the HIV prevention cascade involved a US‐based analysis of Twitter/X posts. NLP was used to identify >10,000 MSM who may be at risk for HIV and who could benefit from targeted PrEP‐related interventions [36]. Geolocator data was also used to determine that 68% of these individuals lived in End the Epidemic priority jurisdictions. Given the global popularity of WhatsApp, Twitter/X and other similar communication formats, NLP has the potential to bring millions into HIV prevention care—a key potential contribution to ending the HIV epidemic as a public health threat.

This analysis is limited by the relatively small dataset, inability to analyse emojis and use in one geographic area with a relatively high‐risk population among those meeting eligibility criteria. Important differences (e.g. lower education, more rural residence, lack of smartphones) were noted between individuals eligible and not eligible for the study. Although the latter had lower HIV risk scores, they may have had less access to PrEP and other HIV prevention services that could not be assessed by our approach. Additionally, funding constraints limited the analysis we could perform. Strengths of the analysis include its innovation, particularly in SSA, and promise as a novel paradigm for identifying unrecognized prevention needs.

## 5 Conclusions

NLP of WhatsApp messages has great potential to find unrecognized HIV prevention needs at scale. Future work should optimize data analytic techniques and test the approach against ground truth (e.g. HIV incidence). The use of passively collected data enables individuals to know more about themselves without the usual limitations of social desirability bias, denial and the need to seek care. Additional research should link those found to have HIV prevention needs with readily available services.

## Author Contributions

JEH, RR and WR conceived the analysis and determined the appropriate methodology. Data curation was performed by BR, VB, SS and RM. JEH, BR, KO, WR and EAB led the investigation in the study, and WR performed validation of the analysis, which was conducted by RR, VB, SS and SM. BR, RR, KO, JBO WR and EAB performed supervision roles. JEH and EAB obtained funding for the study, and EAB was responsible for other resources. RR oversaw data visualization, while KK, JBO and LG oversaw project administration. JEH wrote the original draft of the manuscript; all other authors read and approved the final manuscript.

## Funding

Research reported in this publication was supported by the National Institute of Mental Health United States (R21MH132436).

## Conflicts of Interest

JEH is a paid consultant for Merck. The authors declare no other conflicting financial interests.

## Disclaimer

The content is solely the responsibility of the authors and does not necessarily represent the official views of the National Institutes of Health.

## Figures and Tables

**FIGURE A1:**
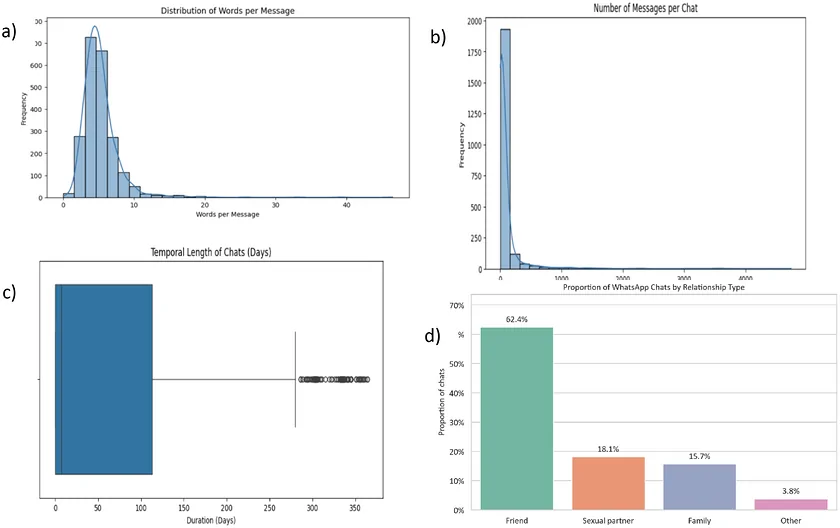
Descriptive visual statistics of WhatsApp datasets per participant, indicating (a) the number of words per message, (b) the number of messages per chat, (c) the duration of a given chat and (d) proportion of chats by relationship type.

**FIGURE A2:**
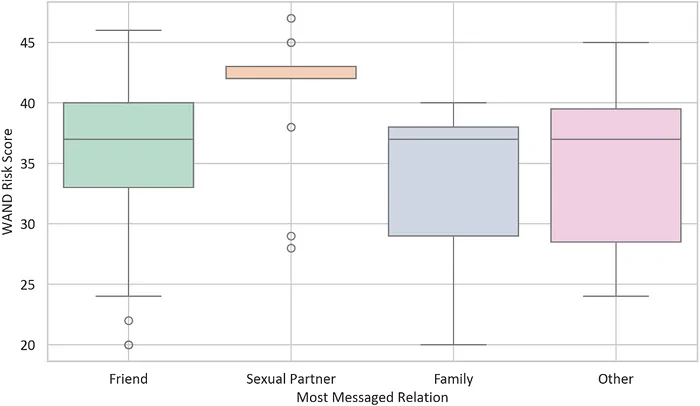
Distribution of the Wand risk score by most messaged relationship (i.e. friend, sexual partner, family member or other).

**FIGURE A3:**
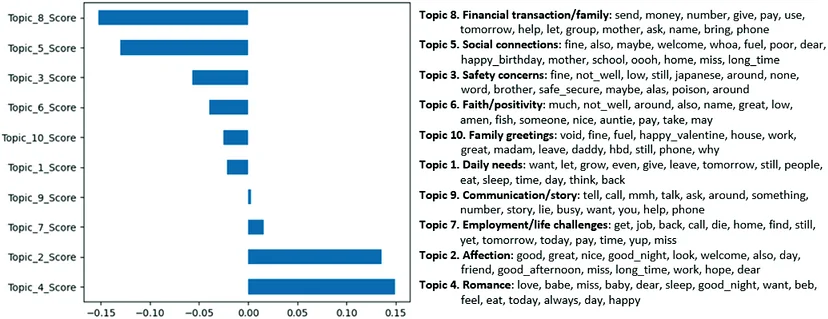
Non‐negative matrix factorization showing correlation between sample topics and the Wand HIV risk score.

**FIGURE A4:**
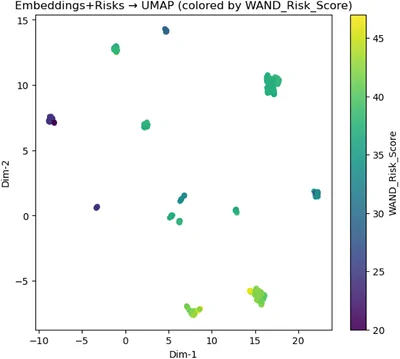
Uniform manifold approximation and projection visualization showing clustering of participants with linguistic similarity as it relates to the Wand risk score. The axes (Dim [dimension] 1 and 2) are the first two dimensions, which represent a compression of each participant's MiniLM sentence embedding. Purple indicates low risk; yellow indicates high risk.

## Data Availability

The data that support the findings of this study are available on request from the corresponding author. The data are not publicly available due to privacy or ethical restrictions.

## APPENDIX A1 Seed Words

### A1.1

Seed words were identified based on preliminary unpublished analyses conducted as a sub‐study within the Monitoring PrEP among Young Adult women study (Haberer et al., JAIDS 2022; 89(3): 251−260), which identified religion words (e.g. amen, god, jesus, church) as associated with a lower VOICE risk score and affectionate words (e.g. beautiful, friend, love, dear) as associated with a higher VOICE risk score. Other seeds were chosen based on input from the study team. Examples of seed words are as follows:

Sexual Seed Words

“abuse, affair, attraction, bedroom, cheek, condom, date, dating, delight, experience, feel, gay, girls, hiv, homosexual, homosexuality, hug, intercourse, intimacy, kiss, kissing, lesbian, love, marriage, nude, partner, partnership, passion, pleasure, pregnancy, prostitution, rape, relationship, romance, sex, sexual, sexuality, sti, teen, test, touch, unsafe”

Religious Seed Words

“allah, angel, belief, bible, bless, christian, church, deity, divine, faith, forgive, god, grace, heaven, holy, jesus, karma, mosque, muslim, pastor, pray, prayer, priest, prophet, quran, religious, resurrection, ritual, sacred, saint, scripture, sermon, sin, spirit, spiritual, temple, worship”

Some exploratory code also used “lexical expansion” tools such as WordNet.
